# Respiratory Syncytial Virus Aggravates Renal Injury through Cytokines and Direct Renal Injury

**DOI:** 10.3389/fcimb.2016.00112

**Published:** 2016-09-30

**Authors:** Songhui Zhai, Lijuan Hu, Lin Zhong, Yannan Guo, Liqun Dong, Ruizhen Jia, Zheng Wang

**Affiliations:** ^1^Department of Pediatrics, West China Second University Hospital, Sichuan UniversityChengdu, China; ^2^Key Laboratory of Birth Defects and Related Diseases of Women and Children (Sichuan University), Ministry of EducationSichuan, China; ^3^Department of Immunology, College of Preclinical and Forensic Medicine, Sichuan UniversityChengdu, China; ^4^West China Institutes of Women and Children's Health, West China Second University Hospital, Sichuan UniversityChengdu, China

**Keywords:** respiratory syncytial virus (RSV), reinfection, renal injury, Immunologic lesion, nephrotic syndrome

## Abstract

The purpose of this study was to investigate the relationship between renal injury and reinfection that is caused by respiratory syncytial virus (RSV) and to analyze the mechanism of renal injury. Rats were repeatedly infected with RSV on days 4, 8, 14, and 28, then sacrificed and examined on day 56 after the primary infection. Renal injury was examined by transmission electron microscopy and histopathology. The F protein of RSV was detected in the renal tissue by indirect immunofluorescence. Proteinuria and urinary glycosaminoglycans (GAGs), serum levels of albumin, urea nitrogen, and creatinine, secretion of cytokines, T lymphocyte population and subsets, and dendritic cell (DC) activation state were examined. The results showed that renal injury was more serious in the reinfection group than in the primary infection group. At a higher infection dose, 6 × 10^6^ PFU, the renal injury was more severe, accompanied by higher levels of proteinuria and urinary GAGs excretion, and lower levels of serum albumin. Podocyte foot effacement was more extensive, and hyperplasia of mesangial cells and proliferation of mesangial matrix were observed. The maturation state of DCs was specific, compared with the primary infection. There was also a decrease in the ratio of CD4^+^ to CD8^+^ T lymphocytes, due to an increase in the percentage of CD8^+^ T lymphocytes and a decrease in the percentage of CD4^+^ T lymphocytes, and a dramatic increase in the levels of IL-6 and IL-17. In terms of the different reinfection times, the day 14 reinfection group yielded the most serious renal injury and the most significant change in immune function. RSV F protein was still expressed in the glomeruli 56 days after RSV infection. Altogether, these results reveal that RSV infection could aggravate renal injury, which might be due to direct renal injury caused by RSV and the inflammatory lesions caused by the anti-virus response induced by RSV.

## Introduction

Nephrotic syndrome (NS) is a nonspecific glomerular disease characterized by proteinuria, which may cause hypoalbuminemia. Minimal change nephrotic syndrome (MCNS) is the most common pathological change in the kidneys of children with NS. Although most children with MCNS were responsive to glucocorticoid treatment, 40–50% patients showed frequent relapses (Hodson et al., 2005). Relapses and aggravation of NS often occur after certain infections (MacDonald et al., 1986; Gulati et al., 2011). In developing countries, 50–70% of relapses reported in children with NS followed upper respiratory tract infection (Arun et al., 2009). Both immunosuppressive agents and increasing maintenance corticosteroid treatment during upper respiratory tract infection were believed to reduce the risk of infection-associated relapses in NS (Mattoo and Mahmoud, 2000; Abeyagunawardena and Trompeter, 2008; Gulati et al., 2011). The mechanisms by which infection induces the relapse of NS might involve up-regulation of cellular immunity and cytokine-mediated inflammation (Kaneko et al., 2002; Bruneau and Dantal, 2009; Zhang et al., 2011). In children with NS, an increase in CD8^+^ T cells and a decrease in the CD4^+^/CD8^+^ ratio were observed at the stage of onset as compared with a normal control group (Daniel et al., 1997; Lama et al., 2002). Infiltrating leukocytes (CD3^+^, CD4^+^, CD8^+^, FoxP3^+^) were found in renal biopsies of children with NS, suggesting that T cells might play an important role in the pathogenesis of idiopathic NS (Benz et al., 2010). Although the differences between steroid-sensitive NS and steroid-resistant NS are controversial, these results indicate that immune disorders may be involved in the pathogenesis of NS. T lymphocytes are activated by pathogens to produce cytokines and chemokines. IL-17, mainly produced by Th17 lymphocytes, play an important role in the onset and progression of renal disease (Matsumoto and Kanmatsuse, 2002, 2003). IL-17 might also contribute to the pathogenesis of NS by decreasing the level of podocalyxin and inducing the apoptosis of podocytes (Shao et al., 2009; Wang et al., 2013). Interleukin-6 (IL-6) is a multifunctional cytokine in the initiation of acute inflammatory responses and is produced by a variety of cells, such as T cells, monocytes, and mesangial cells (Horii et al., 1987; Kishimoto, 1989). IL-6 might play an important role in the pathogenesis of MCNS, and its gene polymorphisms might affect steroid responses in idiopathic nephrotic syndrome (INS) patients (Wang et al., 2010; Jafar et al., 2011). Zachwieja reported that the intracellular synthesis of IL-6 was decreased in NS (Zachwieja et al., 2002). TGF-β may promote mesangial cell dysfunction and may be involved in the refined balance between survival and apoptotic responses in podocytes (Kato et al., 2006). Dendritic cells (DCs), a class of antigen-presenting cells, are important for balancing immune responses and tolerance (Banchereau and Steinman, 1998; Raimondi et al., 2007). Upon antigen capture, immature DCs highly express MHC class II molecules and CD86 molecules, and become mature DCs. DCs play important roles in both the development of renal inflammation and the pathophysiology of glomerulonephritis (Kurts et al., 2007; Matsumoto et al., 2010). However, it is unknown whether DCs play any role in pathogenesis of primary NS.

In 1986, MacDonald et al. first reported that respiratory syncytial virus (RSV) could aggravate primary NS (MacDonald et al., 1986). Our previous studies showed that RSV was the most commonly identified respiratory tract virus in children with steroid-sensitive and simple NS (Wang et al., 1999, 2001; Liu et al., 2006). Kawasaki et al. found that the severity of illness induced by RSV reinfection was generally milder than that induced by the RSV primary infection (Kawasaki et al., 2004). In Kenya, a study in a children's clinic found that immunity to RSV reinfection was partial and short-lived (Ohuma et al., 2012). But in animal models of RSV infection, the role of T cell responses in exacerbated disease was implicated (Sacco et al., 2015). Tregoning et al. reported that T cells, in particular CD8^+^ T cells, play a central role in defending against neonatal RSV infection and could exacerbate disease during RSV reinfection (Tregoning et al., 2008). Mature DCs could activate naïve T cells and play crucial roles in anti-RSV immune responses (Farrag and Almajhdi, 2016).

RSV infection could induce the secretion of cytokines, such as IL-6, IL-13, and IL-17 (Johnson and Graham, 1999; Guerrero-Plata et al., 2001). Previously, using a rat model of RSV infection, we observed elimination of RSV at the later stage of infection (after 28 days), as well as an increase in urine protein secretion, indicating that the large amount of urine protein secretion during infection-induced kidney injury might be related to immune dysfunction (Liu et al., 2007). In other words, both the direct damage and the dysregulation of the immune response upon RSV infection might contribute to renal damage.

In this study, using the rat model of RSV reinfection, we aimed to investigate the mechanism by which RSV reinfection aggravates renal injury. Is it by direct damage of glomerular basement membrane or by dysregulation of immune responses.

## Materials and methods

### Viruses and cells

The Long strain of Human RSV and HeLa cell line were obtained from the Viral Institute of the Chinese Academy of Preventive Medical Science. RSV was cultured in HeLa cells and assayed for infectivity (Liu et al., 2007). HeLa cells were cultured in Dulbecco's modified Eagle's medium (DMEM; Gibco Invitrogen, Carlsbad, CA) containing 10% fetal calf serum at 37°C, 5% CO_2_. RSV was propagated in HeLa cells in a monolayer culture. RSV titer was determined by a methylcellulose plaque assay.

### Animals

Male Sprague-Dawley rats (age: 6–7 weeks, weight: 180–200 g), free of specific pathogens (Da Shuo Company, Sichuan, China), were randomly allocated into four groups (Group A: 6 × 10^4^ PFU primary infection group, *n* = 20); Group B: 6 × 10^6^ PFU RSV reinfection group, *n* = 20); Group C: 6 × 10^4^ PFU RSV reinfection group, *n* = 20); Group D: the mock infection control group, *n* = 20). First, Groups A, B, and C were all inoculated with 6 × 10^4^ PFU RSV intranasally (0.2 ml) and intraperitoneally (0.4 ml) daily for 3 continuous days, whereas Group D was inoculated with virus-free DMEM intranasally (0.2 ml) and intraperitoneally (0.4 ml). On day 4, 8, 14, or 28, Groups A and D were re-inoculated with virus-free DMEM intranasally (0.2 ml) and intraperitoneally (0.4 ml) daily for 3 continuous days, respectively, defined as A1, A2, A3, A4 and D1, D2, D3, D4, respectively. On day 4, 8, 14, or 28, Group B was re-inoculated with 6 × 10^6^ PFU intranasally (0.2 ml) and intraperitoneally (0.4 ml) daily for 3 continuous days, defined as B1, B2, B3, and B4. Group C was re-inoculated with 6 × 10^4^ PFU RSV in the same way, defined as C1, C2, C3, and C4. Each subgroup consisted of 5 rats (*n* = 5).

On the day after each re-inoculation, 24 h urine samples were collected. All rats were sacrificed on day 56. Blood samples were collected from the heart and then examined.

All animal experimentation was approved by the Ethics Committee of Sichuan University. Institutional and Government Review Boards approved all animal studies.

### Measurement of proteinuria, urinary glycosaminoglycans (GAGs) excretion, and serum parameters

The amount of the urinary protein was measured by pyrogallol end-point method, while urinary GAGs was examined by modified Whiteman process (Hotz et al., 1991; Liu et al., 2007). The urinary GAGs were calculated using a calibration curve, with heparan sulfate as standard, and were corrected with urinary creatinine. Serum levels of albumin, urea nitrogen, and creatinine were measured using a Hitachi 7600 machine (Hitachi 7600; Hitachi, Tokyo, Japan).

### Transmission electron microscopy and histopathology of the kidney

The shape and the weight of the whole kidney were first recorded. Then, renal tissue (0.5 × 0.7 cm) was fixed in 4% paraformaldehyde at 4°C for 24 h, then embedded in paraffin. Paraffin sections (4 μm) were used for hematoxylin and eosin staining. Fresh renal tissue was fixed in 3% glutaraldehyde phosphate buffer for ultrastructural analysis by electron microscopy (H-600IV transmission electron microscope, Hitachi).

### Indirect immunofluorescence of renal tissue

Renal tissue (0.5 × 0.7 cm) was fixed in 4% paraformaldehyde at 4°C for 24 h and embedded in paraffin. Paraffin sections (4 μm) were used to detect RSV F protein using anti-respiratory syncytial virus F glycoprotein antibody (Abcam company, US) via indirect immunofluorescence.

### Measurement of cytokines secretion

The serum levels of IL-6 and IL-17 were examined by enzyme-linked immunosorbent assay (ELISA) (R&D System, Minneapolis, MN). Each sample was assayed in triplicate.

### Measurement of CD4^+^ T and CD8^+^ T cells

Peripheral blood mononuclear cells (PBMCs) were obtained from heparinized peripheral blood of subjects by Ficoll-Hypaque density centrifugation. The cells were centrifuged at 900 × g for 20 min. Then, pellets were washed with 0.01 M PBS and then re-suspended in 0.01 M PBS supplemented with 5% FBS (about 10^6^ cells/mL). One hundred microliters cell suspension were incubated with eFluor450 anti-rat CD3, phycoerythrin (PE)-Cy7 anti-rat CD8, and anti-rat CD4 (5 μL, antibody/0.01 M PBS: 1:10) (BioLegend, San Diego, CA), or respective isotype controls for 15 min at 4°C. Multicolor flow cytometry was performed on a Becton Dickinson FACScalibur® (BD, San Jose, USA). Each specimen was analyzed in triplicate.

### Identification of Ox62, MHCII, and CD86 on dendritic cells

Dendritic cells (DCs) were isolated and cultured using previously described methods with some modification (Mayordomo et al., 1997; Qiao et al., 2007). Briefly, the bone marrow mononuclear cells (BMMCs) were obtained from the bone marrow of rat limbs by density gradient centrifugation. DCs were then obtained from BMMC culture in RPMI 1640 medium supplemented with 10% fetal calf serum (Gibco Invitrogen, Carlsbad, CA) recombinant rat granulocyte-macrophage colony-stimulating factor (rrGM-CSF, 10 ng/ml, Pepro Tech, Rocky Hill, NJ), and recombinant rat interleukin-4 (rrIL-4, 10 ng/ml, Pepro Tech). After about 6–7 days, the interphase cells were harvested. The pellets were washed with 0.01 M PBS and then re-suspended in 0.01 M PBS supplemented with 5% FBS (about 2 ~ 3 × 10^6^ cells/mL). One hundred microliters of DC suspension were incubated with fluorochrome-labeled monoclonal antibodies specific for PE-OX62, MHC-II, CD86 (5 μL, antibody/0.01 M PBS: 1:10) (BioLegend, San Diego, CA) and stained for 15 min on ice. Multicolor flow cytometry was performed on a Becton Dickinson FACScalibur®. Every sample was analyzed in triplicate.

### Statistical analysis

Statistical analyses were performed using the Statistical Package for Social Sciences ver16.0. Data are expressed as mean ± SD. For multiple comparisons, the Bonferroni or Dunnett T3 tests were used. *P* < 0.05 was considered significant.

## Results

### RSV reinfection resulted in enhanced proteinuria

**Figure 1:**
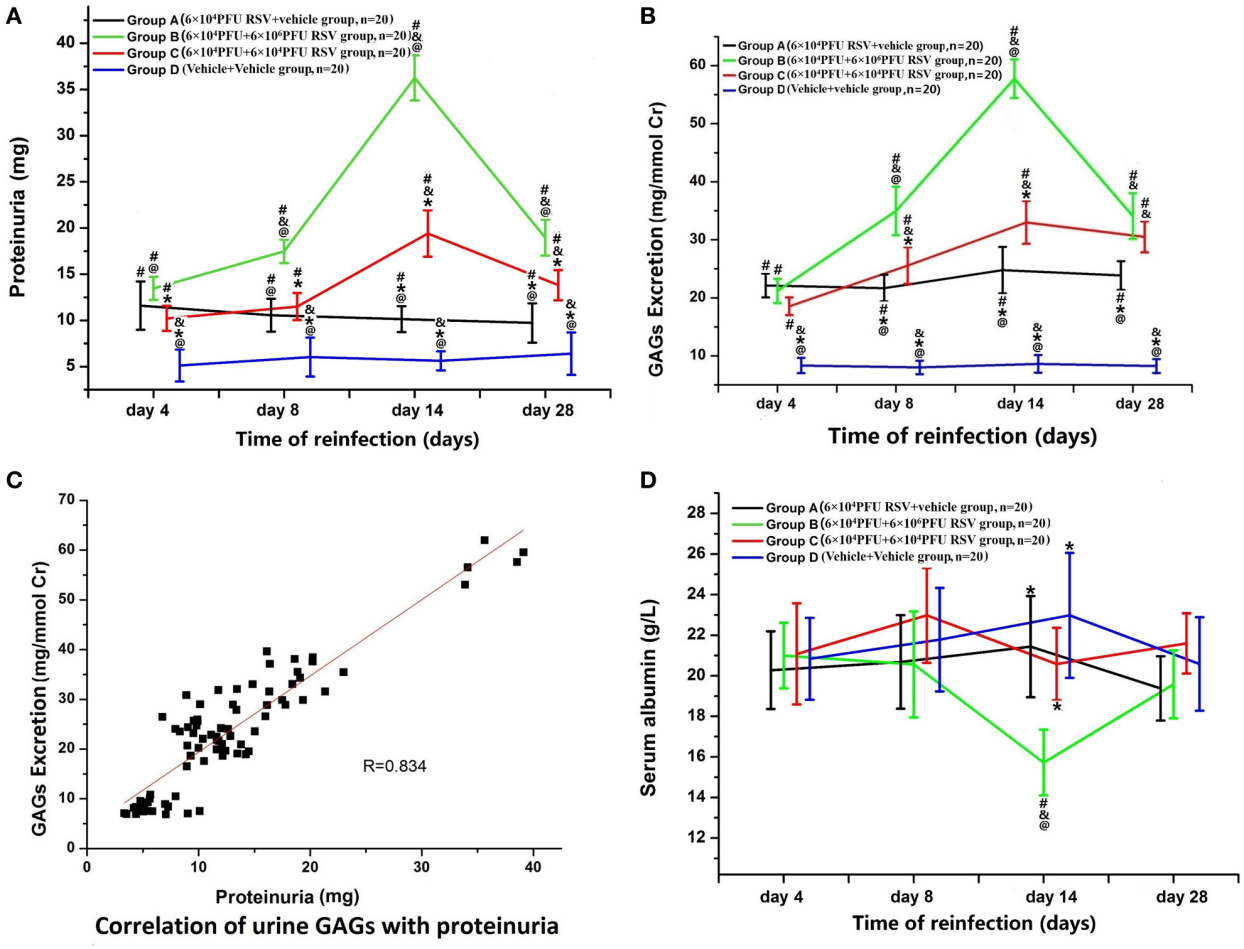
**(A)** Changes in proteinuria. Proteinuria was increased in Group A (*n* = 20), Group B (*n* = 20), and Group C (*n* = 20) (*P* < 0.05). The proteinuria in Groups B and C was higher than that in Group A (*P* < 0.05), which indicates that both RSV infection and reinfection could result in significantly increased proteinuria. Among different time points of re-inoculation, the proteinuria on the day 14 was the highest (*P* < 0.05). ^#^*P* < 0.05, vs. Group D; ^&^*P* < 0.05, vs. Group A; ^*^P < 0.05, vs. Group B; ^@^*P* < 0.05, vs. Group C; blank, *P* > 0.05, vs. any corresponding groups. **(B)** Changes in urinary GAGs. 2A: Urinary GAG excretion was elevated in Group A (*n* = 20), Group B (*n* = 20), and Group C (*n* = 20) (*P* < 0.05). The urinary GAG excretion in Groups B and C was higher than that in Group A on the days 8, 14, and 28 (*P* < 0.05). Urinary GAG excretion in Group B was higher than that in Group C on the day 8 and 14 subgroups (*P* < 0.05). ^#^*P* < 0.05, vs. Group D; ^&^*P* < 0.05, vs. Group A; ^*^P < 0.05, vs. Group B; ^@^*P* < 0.05, vs. Group C; blank, *P* > 0.05, vs. any corresponding groups. **(C)** Urinary GAG excretion were positively correlated with proteinuria (*R* = 0.834, *P* < 0.05). **(D)** Changes in serum albumin. The serum level of albumin in the Group B, day 14 subgroup was significantly decreased compared with the other three groups (*P* < 0.05). ^#^*P* < 0.05, vs. Group D; ^&^*P* < 0.05, vs. Group A; ^*^P < 0.05, vs. Group B; ^@^*P* < 0.05, vs. Group C; blank, *P* > 0.05, vs. any corresponding groups.

As shown in Figure 1A, increased proteinuria was found in Group A (*n* = 20), Group B (*n* = 20), and Group C (*n* = 20), compared with Group D (*n* = 20). This indicates that both RSV infection and reinfection could result in a significant increase in proteinuria (*P* < 0.05). Compared with Group A, an increase in protein excretion was found in both Group B and Group C to some extent, and this increase was positively associated with RSV reinfection titer (*P* < 0.05).

Variable levels of proteinuria were found at different time points of re-inoculation. Group B3 (*n* = 5) and Group C3 (*n* = 5) yielded the highest level of proteinuria among different time points within their groups. Excretion levels in Group B was significantly higher than those in the other three groups (*P* < 0.05; Figure 1A).

As in Figure 1B, urinary GAG excretion in Groups A, B, and C was significantly higher than in Group D (*P* < 0.05). Urinary GAG excretion in both Groups B and C were higher than in Group A, especially in Group B2 (*n* = 5), B3, B4 (*n* = 5), C2 (*n* = 5), C3 and C4 (*n* = 5) (B2, C2 vs. A2; B3, C3 vs. A3; B4, C4 vs. A4, *P* < 0.05, *n* = 5 each). The highest amounts of urinary GAG excretion were found in Groups B3 and C3 among different time points (*P* < 0.05; Figure 1B). Furthermore, as shown in Figure 1C, urinary GAGs and proteinuria were positively correlated (*R* = 0.834, *P* < 0.05).

### RSV reinfection resulted in enhanced hypoalbuminemia

The serum level of albumin in Group B3 (*n* = 5) was significantly decreased compared with the other three groups (*P* < 0.05; Figure 1D), while there were no significant differences in the levels of blood urea nitrogen or serum creatinine among the groups (data not shown).

### Histopathology in kidney

#### Light microscopy reveals aggravated renal damage in RSV reinfection

**Figure 2:**
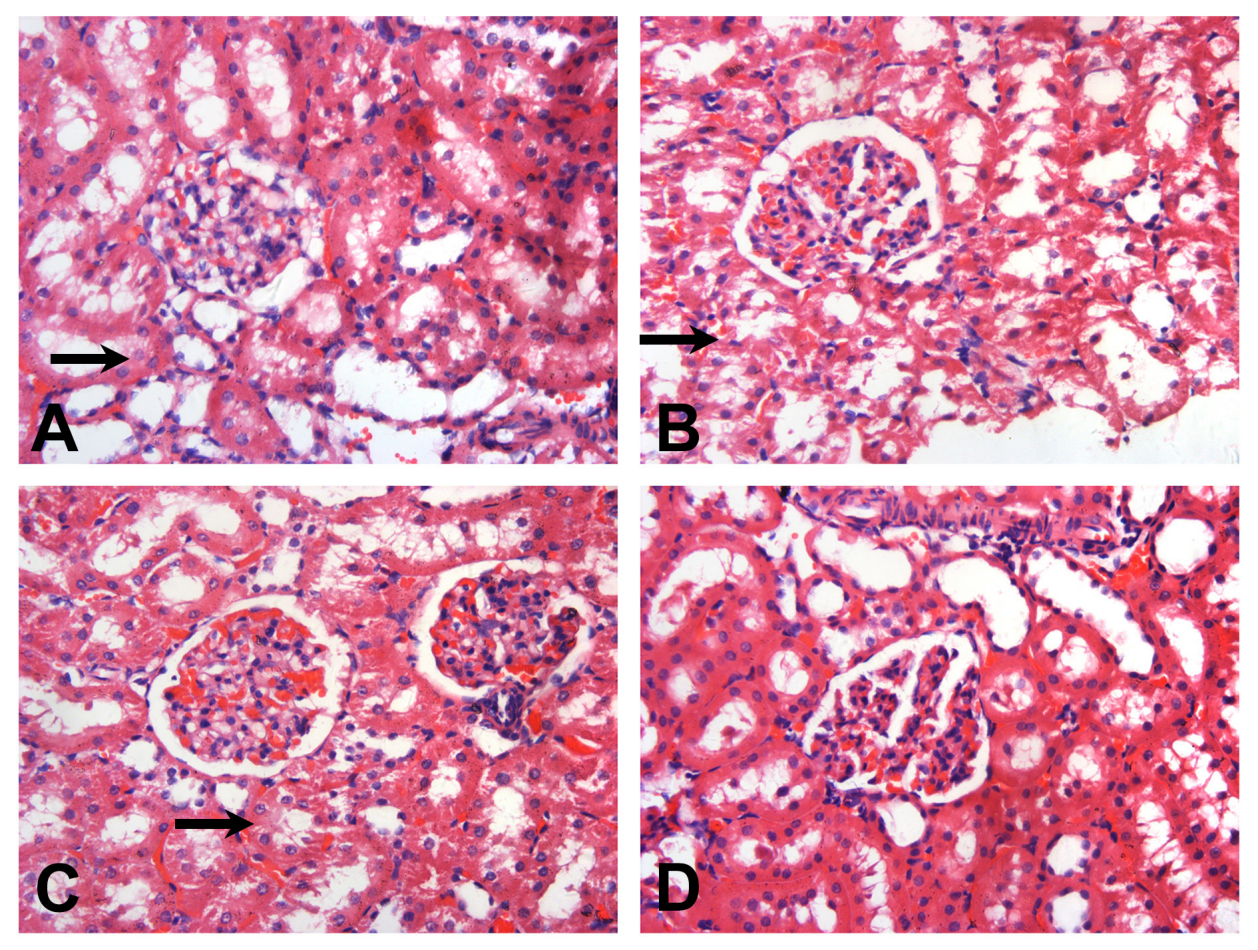
**Representative sections of the glomeruli of SD rats on the day 14 (HE, × 200)**. After RSV reinfection, the renal tubules were shown with different extents of vacuolar degeneration (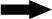). The renal interstitium was swollen. The changes in Groups B3 **(B)** and C3 **(C)** were more serious than that in Group A3 **(A)**. There were hardly obvious changes on the renal tubules in Group D3 **(D)**. No obvious changes were observed in the glomeruli under light microscopy. **(A)** The 6 × 10^4^ PFU, RSV + vehicle, day 14 subgroup (Group A3); **(B)** The 6 × 10^4^ PFU RSV + 6 × 10^6^ PFU RSV, day 14 subgroup (Group B3); **(C)** The 6 × 10^4^ PFU RSV + 6 × 10^4^ PFU RSV, day 14 subgroup (Group C3); **(D)** Vehicle + vehicle group, day 14 subgroup (Group D3).

The histopathology of the kidney was similar in all subgroups; thus, representative figures of one out of twenty rats were are demonstrated. Compared with Group D3 (Figure 2D), in Group B3 (Figure 2B) and Group C3 (Figure 2C), renal tubules were diffusely vacuolated with granular degeneration, while the renal interstitium was expanded by edema. In Group A3 (Figure 2A), mild granular degeneration was observed in tubular epithelial cells. No obvious change was observed in the glomeruli under light microscopy in all RSV infection groups (Figure 2).

#### Electron microscopy reveals aggravated foot-process fusion in RSV reinfection

**Figure 3:**
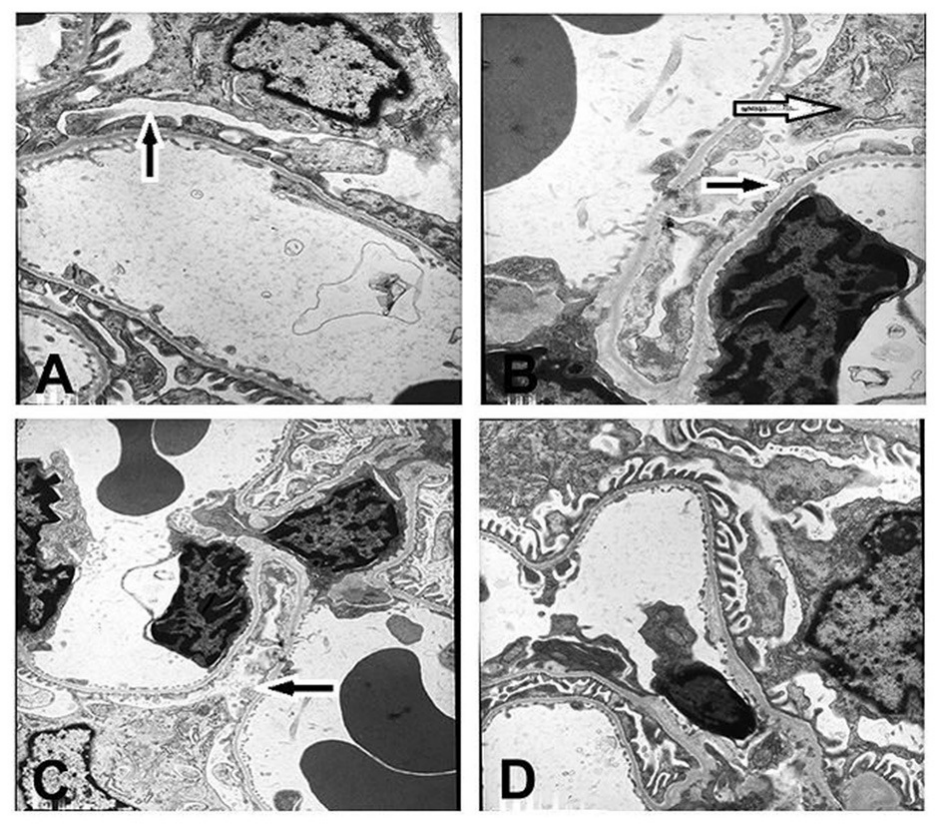
**Ultrastructure of renal tissue (× 6000)**. Podocytes had undergone foot process fusion to different extents **(A–C)**. There was some mesangial cell and mesangial matrix proliferation in B. No electron-dense deposits were observed in **(D). (A)** The 6 × 10^4^ PFU RSV + vehicle, day 14 subgroup (Group A3); **(B)** The 6 × 10^4^ PFU RSV + 6 × 10^6^ PFU RSV, day 14 subgroup (Group B3); **(C)** The 6 × 10^4^ PFU RSV + 6 × 10^4^ PFU RSV day 14 subgroup (Group C3); **(D)** Vehicle + vehicle group, day 14 subgroup (Group D3). Black arrow (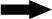) represents the foot profusion of podocytes, and the white arrow (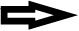) shows the mesangial matrix proliferation.

In Group A3, segmental foot-process effacement of glomerular epithelial cells was observed (~30%; Figure 3A). In Group B3, foot-processes of the podocytes were universally fused or lost, accompanied by mesangial cell, and matrix proliferation (Figure 3B). Compared with Group A3, foot-process fusion was also aggravated in Group C3 (Figure 3C), but was hardly observed in Group D3 (Figure 3D). No electron-dense deposits were observed in any of the groups. Again, since the histopathology of kidney was similar in all subgroups, only representative figures of one out of twenty rats are shown (Figure 3).

### RSV F protein is present in the rat kidney

**Figure 4:**
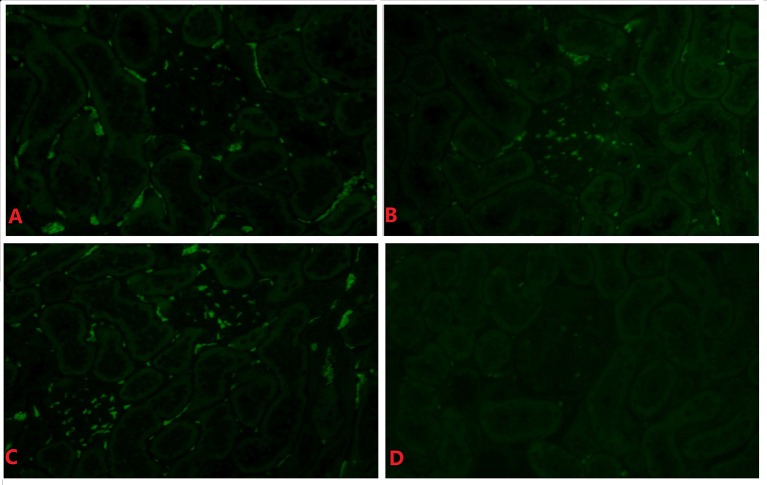
**RSV F protein in renal tissue of the day 14 subgroup (indirect immunofluorescence, × 400)**. Positive hybridization signals appeared in the glomeruli and tubules of the RSV infection group in **(A–C)**. There was little positive expression of RSV F protein in **(D). (A)** The 6 × 10^4^ PFU RSV + vehicle, day 14 subgroup (Group A3); **(B)** The 6 × 10^4^ PFU RSV + 6 × 10^6^ PFU RSV, day 14 subgroup (Group B3); **(C)** The 6 × 10^4^ PFU RSV + 6 × 10^4^ PFU RSV, day 14 subgroup (Group C3); **(D)** Vehicle + vehicle group, day 14 subgroup (Group D3).

Representative figures of one out of twenty rats are shown. RSV F protein was detected in renal tissues in all RSV infection groups, especially during the late stage of infection (Figures 4A–C). F protein was mainly detected in the glomeruli but was seldomly in the renal interstitium in all infected animals and not detected in the negative control animals (Figure 4D).

### RSV reinfection resulted in enhanced serum IL-6 and IL-17 levels

**Figure 5:**
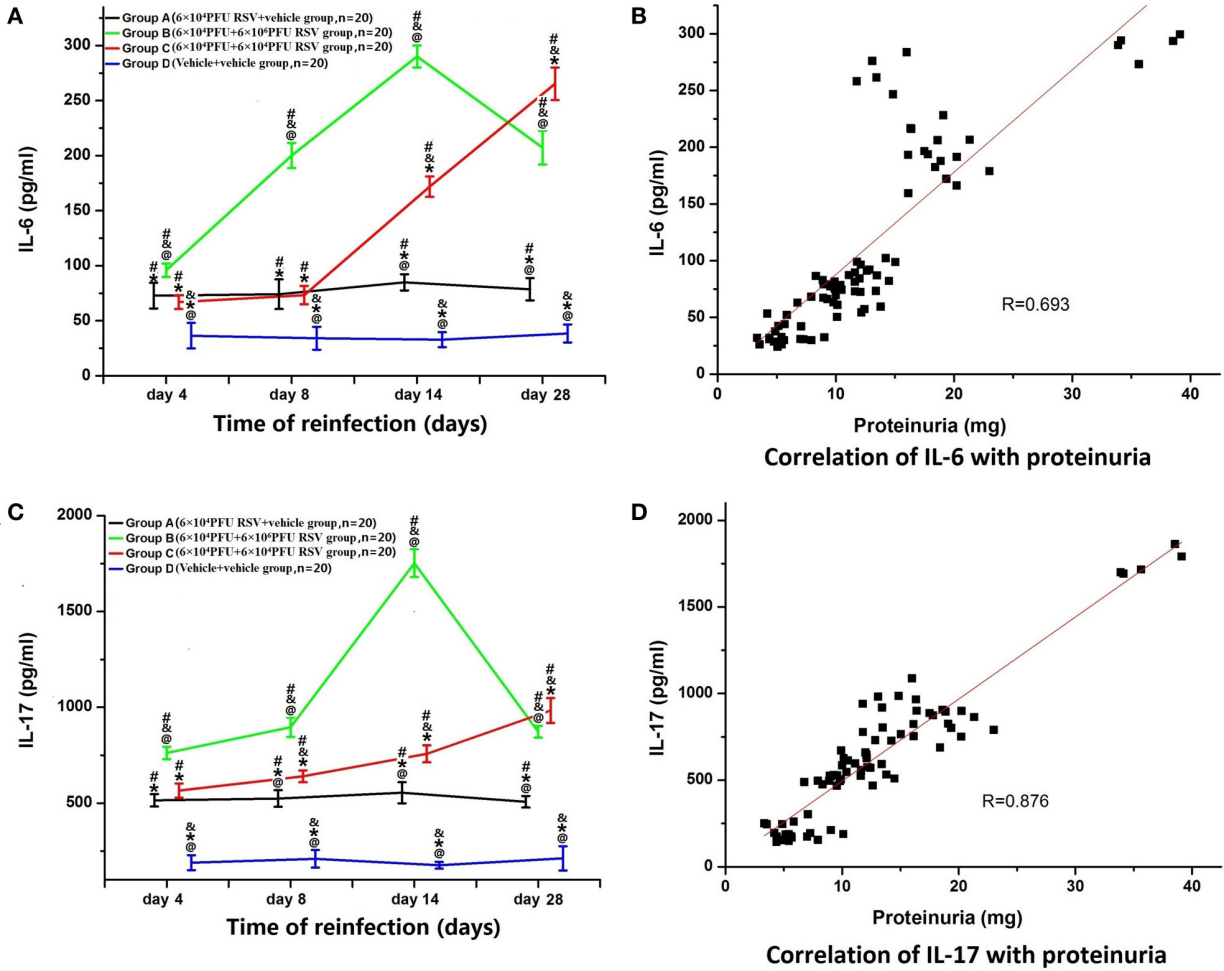
**Serum levels of IL-6 and IL-17 and their relationships with proteinuria. (A)** The levels of serum IL-6 in Groups A (*n* = 20), B (*n* = 20), and C (*n* = 20) were all significantly higher than that in Group D (*n* = 20). The level of IL-6 in the Group B was higher than that in Groups A and C. Compared with Group A, the level of IL-6 was also elevated in Group C. ^#^*P* < 0.05, vs. Group D; ^&^*P* < 0.05, vs. Group A; ^*^P < 0.05, vs. Group B; ^@^*P* < 0.05, vs. Group C; blank, *P* > 0.05, vs. any corresponding groups. **(B)** The level of serum IL-6 was positively correlated with proteinuria (*R* = 0.693, *P* < 0.05). **(C)** Serum levels of IL-17 and the relationship with proteinuria. **(A)** The levels of serum IL-17 in Groups A (*n* = 20), B (*n* = 20), and C (*n* = 20) were all significantly higher than that in Group D (*n* = 20). The level of IL-17 in Group B was higher than that in Groups A and C. Compared with Group A, the level of IL-17 was also increased in Group C. ^#^*P* < 0.05, vs. Group D; ^&^*P* < 0.05, vs. Group A; ^*^P < 0.05, vs. Group B; ^@^*P* < 0.05, vs. Group C; blank, *P* > 0.05, vs. any corresponding groups. **(D)** The level of serum IL-17 was positively correlated with proteinuria (*R* = 0.876, *P* < 0.05).

The levels of serum IL-6 in Group A (*n* = 20), B (*n* = 20), and C (*n* = 20) were significantly higher than that in Group D (*n* = 20; *P* < 0.05). Compared with Group A, the levels of IL-6 in Group B were significantly higher between subgroups (B1 vs. A1, B2 vs. A2, B3 vs. A3, B4 vs. A4; *P* < 0.05). In Group C, only the IL-6 levels in Groups C3 and C4 were significantly higher than that in Groups A3 and A4 (C3 vs. A3, C4 vs. A4; *P* < 0.05). The serum IL-6 level in Group B3 was the highest among all Group B time points, whereas the IL-6 level in Group C4 was the highest of Group C. Compared with Group C, serum IL-6 levels were significantly higher in Group B (B2 vs. C2, B3 vs. C3, B4 vs. C4), except for the day 4 time point (B1 vs. C1) (*P* < 0.05; Figure 5A). Moreover, serum IL-6 levels were positively correlated with proteinuria (*R* = 0.693, *P* < 0.05; Figure 5B).

Similarly, the levels of serum IL-17 in Groups A (*n* = 20), B (*n* = 20), and C (*n* = 20) were all significantly higher than those in Group D (*n* = 20) (*P* < 0.05). The IL-17 levels in Group B at all time points were significantly higher than in Group A (B1 vs. A1, B2 vs. A2, B3 vs. A3, B4 vs. A4; *P* < 0.05). In Group C, IL-17 levels were significantly higher only in Groups C2, C3, and C4, compared with corresponding time points in Group A (C2 vs. A2, C3 vs. A3, C4 vs. A4; *P* < 0.05). The serum IL-17 level in Group B3 was the highest among all of Group B, whereas the serum IL-17 level in Group C4 was the highest among all Group C. Compared with Group C, serum IL-17 levels were significantly elevated in Groups B2, B3, and B4 (B2 vs. C2, B3 vs. C3, B4 vs. C4) (*P* < 0.05; Figure 5C). Moreover, the levels of serum IL-17 were positively correlated with proteinuria (*R* = 0.876, *P* < 0.05; Figure 5D).

### RSV reinfection aggravated the imbalance of CD4^+^/CD8^+^ T lymphocytes

**Figure 6:**
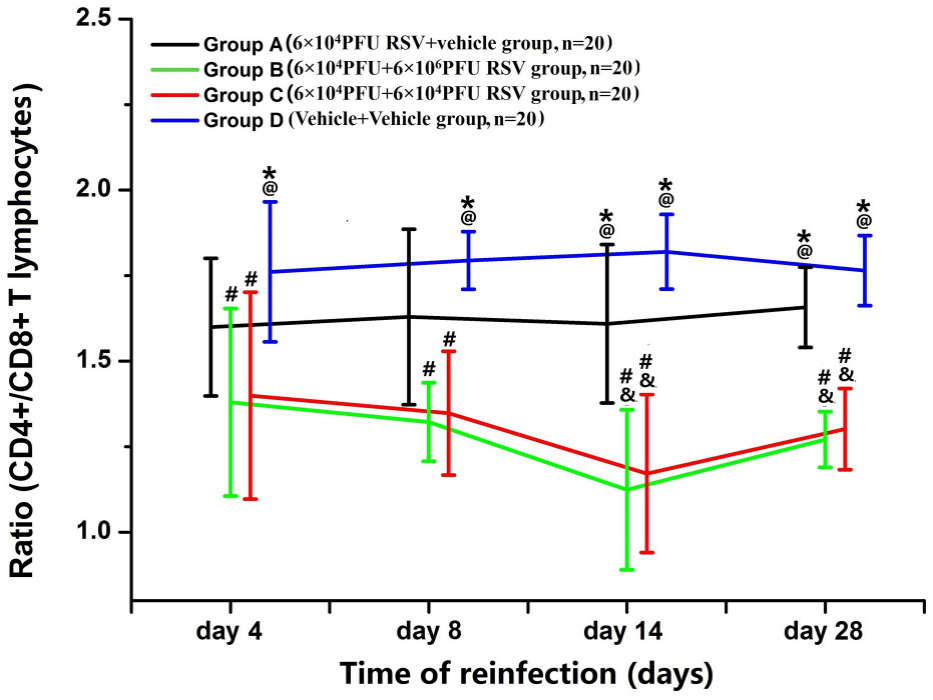
**Ratio of CD4^+^/CD8^+^ T lymphocytes**. Compared with Group A (*n* = 20), the ratio of CD4^+^/CD8^+^ in Group B (*n* = 20) and Group C (*n* = 20) was all decreased to some extent. ^#^*P* < 0.05, vs. Group D; ^&^*P* < 0.05, vs. Group A; ^*^P < 0.05, vs. Group B; ^@^*P* < 0.05, vs. Group C; blank, *P* > 0.05, vs. any corresponding groups.

The ratio of CD4^+^ T lymphocytes to CD8^+^ T lymphocytes (CD4^+^/CD8^+^) was significantly decreased in Groups B (*n* = 20) and C (*n* = 20), compared with Group D (*n* = 20) (*P* < 0.05). Compared with Group A (*n* = 20), the ratio of CD4^+^/CD8^+^ in both Groups B and C was reduced to some extent. The ratios in Groups B3, B4, C3, and C4 were all significantly decreased, compared with Groups A3 and A4 (*P* < 0.05). The ratio was not statistically different between Groups B and C (*P* > 0.05; Figure 6). Compared with Groups D and A, the percentages of CD4^+^ T lymphocytes were reduced in Groups B and C (*P* < 0.05). There were no significant differences between Groups B and C (*P* > 0.05; data not shown). The percentages of CD8^+^ T lymphocytes were elevated in Groups B3 and C3 compared with those in Group A3 and D3 (*P* < 0.05). The percentage of CD8^+^ T lymphocytes was not significantly different between Groups B3 and Group C3 (*P* > 0.05; data not shown).

### RSV reinfection resulted in the maturation of DCS from BMMC

**Figure 7:**
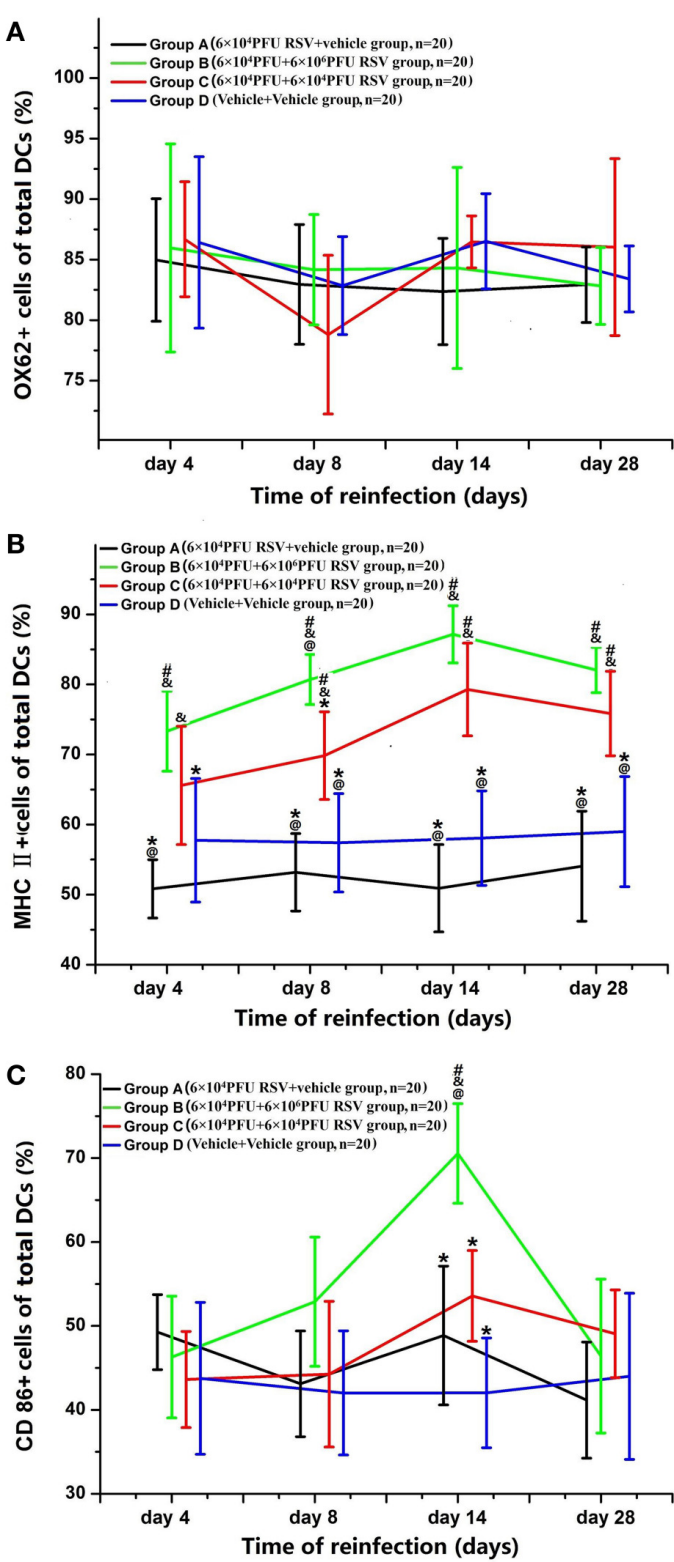
**Changes in the surface markers of DCs from BMMC**. The percentage of OX62^+^ cells in the DCs showed no significant differences between the four groups **(A)**. The percentage of MHC II^+^ cells amongst the DCs was greater in Groups B (*n* = 20) and C (*n* = 20) than in Groups A (*n* = 20) and D (*n* = 20) on the day 8, 14, and 28 **(B)**. The percentage of CD86^+^ cells amongst the DCs was dramatically increased in Groups B and C **(C)**. ^#^*P* < 0.05, vs. Group D; ^&^*P* < 0.05, vs. Group A; ^*^P < 0.05, vs. Group B; ^@^*P* < 0.05, vs. Group C; blank, *P* > 0.05, vs. any corresponding groups.

As shown in Figure 7, the percentage of OX62^+^, MHC II^+^, and CD86^+^ cells in all DCs developed from BMMCs were counted. The percentage of OX62^+^ cells amongst the DCs showed no significant difference between the four groups (*P* > 0.05; Figure 7A). In Figure 7B, the percentages of MHC II^+^ cells amongst the DCs were dramatically increased on days 8, 14, and 28 in Groups B and C, compared with Groups A and D (*P* < 0.05). The percentages of MHC II^+^ cells amongst the DCs in Groups B3 and C3 were the highest among all time points in their respective groups (*P* < 0.05). There was no difference in the percentage of MHC II^+^ cells in the DCs between Groups B and Group C, except between the day 8 subgroups (*P* > 0.05). As demonstrated in Figure 7C, the percentage of CD86^+^ cells in all DCs was dramatically increased in Group B3 among all of the subgroups (*P* < 0.05). The total number of DCs was similar, and the percentages of both CD86^+^ and MHC II^+^ cells amongst the DCs were elevated, suggesting that DCs undergo maturation upon RSV reinfection (Figure 7).

## Discussion

In previous clinical observations, we found that the onset and relapse of NS was related to infection. Using a rat model, we found that RSV could induce proteinuria, with extensive effacement of the foot processes of glomerular epithelial cells; yet few changes in mesengial cells or renal tubular epithelial cells were observed (Liu et al., 2007). However, whether RSV reinfection could aggravate proteinuria and renal injury was still unknown.

Since the titer of RSV in the nasopharynx varies from 10^3.5^ TCID to 10^6^ TCID during the respiratory infection of children, 6 × 10^4^ and 6 × 10^6^ PFU RSV suspension were selected for reinfection experiments in rats. In this research, we found that 6 × 10^4^ PFU RSV primary infection could result in obvious proteinuria but not reduced levels of serum albumin or extensive effacement of foot process in the glomeruli. This result was consistent with Liu's study (Liu et al., 2007). Moreover, the RSV reinfection group could aggravate the pathological changes in the kidney, accompanied by a notable decrease in serum albumin level. Yet the levels of serum urea nitrogen and creatinine were still normal (results were not shown). RSV reinfection with higher titers induced more serious damage. These results suggest that RSV infection could induce the renal injury, and RSV reinfection aggravates the renal damage. However, the underlying mechanism is largely unknown.

Firstly, we propose that RSV infection may induce renal injury by directly destroying the glomerular filtration barrier. Compared with the uninfected control group, the primary infection group and the reinfection groups showed a dramatically greater increase in proteinuria and urinary GAG levels, which suggested the electric barrier of glomerular basement membrane were injuried. There was negatively-charged GAGs chains on glomerular basement membrane. In previous studies, Hallak et al. and Schmidtke et al. showed that cell surface GAGs, heparan sulfates, were necessary for RSV infection (Hallak et al., 2000; Schmidtke et al., 2003). Moreover, Guo et al. showed that in rats, low-molecular weight heparin could alleviate proteinuria by preventing RSV from binding to HS (Guo et al., 2008). These reports suggested that RSV might induce renal injury by directly destroying the glomerular basement membrane. In our study, the detection of RSV F protein in the kidney further supports the notion that RSV may cause direct kidney damage. However, we found this at the late stage of RSV infection (28 days and later); proteinuria did not completely conform with RSV expression or urinary GAG excretion, which suggests that alternative mechanisms might take place in the process of rat nephropathy induced by RSV infection.

In this study, an increase in the percentages of MHC-II^+^ and CD86^+^ cells in BMMC was observed when compared with the control groups, which suggest that DCs undergo maturation upon RSV infection. Mature DCs are more effective in antigen presentation to activate T-cells (Jones et al., 2006; Lindell et al., 2008). At an early stage of RSV infection, pulmonary DCs have an important role on anti-virus immune surveillance. And at late stages, DCs induce cellular immunity (Usharauli, 2005; Zhang et al., 2016.). This study showed that the ratio of CD4^+^/CD8^+^ T lymphocytes was abnormal. The percentage of CD8^+^ T lymphocytes was elevated, while the percentage of CD4^+^ T lymphocytes was reduced, especially in RSV reinfection. This suggests that RSV reinfection increases DC maturation, subsequently resulting in a disorder of immune function.

These results indicate that there is immune dysregulation after RSV infection, and RSV reinfection could aggravate this phenomenon. In renal sections, however, massive infiltration of lymphocytes was not observed. This observation is consistent with some previous animal and clinic trials with nephrotic syndrome (Daniel et al., 1997; Lama et al., 2002). These results suggest that mature DCs might induce T lymphocyte activation and abnormal differentiation of T lymphocytes, which may induce the immune disorder and the increase of pro-inflammatory cytokines.

During host defense against RSV infection, a number of cytokines are secreted, including type I IFN, IFN-γ (Durbin et al., 2002; Spann et al., 2005; Ramaswamy et al., 2006), IL-4, IL-6, CCL3, CCL2, and CCL5 (Hornsleth et al., 2001; Welliver et al., 2002; Mellow et al., 2004). Some cytokines, such as IFNs, can directly contribute to the clearance of viruses, while some cytokines might also contribute to organ injury. Serum levels of IL-6 and IL-17 were elevated following RSV infection, and were higher following RSV reinfection than primary infection. When the cytokines circulate through the kidney, they may bind with their receptors and subsequently induce kidney injury. On the other hand, the micro-circumstance of the kidney, which showed alterations, might contribute to the aggravation of renal damage. All of these results imply that immune dysfunction might play an important role in the pathogenesis of renal injury in addition to the direct effects of RSV.

On the other hand, cytokines such as IL-17 and IL-6 could bind with their receptors on mesangial cells and renal tubular epithelial cells, leading to the release of many chemokines that can recruit neutrophils and monocytes to the kidney. This process can trigger injury to the kidney (Turner et al., 2010). Yet in this study, a leukocytic infiltrate was not observed in the renal sections, suggesting that there might be other mechanisms by which IL-17 and IL-6 damage the kidney.

Generally, during the primary immune response (e.g., to RSV), the immune system is activated after a long lag-phase and then declines to a new balanced condition. During the secondary immune response, a faster, stronger, and more effective, secondary immune response is mediated by memory lymphocytes (Lee et al., 2015). Therefore, after an acute pulmonary infection with RSV, the virus-specific memory T-cells can persist in the lungs for a long time. The amplification of memory T cells appears to be the most effective in organized lymphoid tissues (Ostler et al., 2001). In addition, through studies in clinical trials and animal models, it was found that severe bronchiolitis induced by RSV infection was associated with recurrent wheezing and chronic obstructive pulmonary disease, which might be related to immune disturbance (Guerrero-Plata et al., 2001). Hou reported that Th17 cells play an essential role in persistent viral infection (Hou et al., 2009). Here, our study shows that RSV F protein was still observed in the renal tissues in the late stage of RSV infection, and that the level of IL-17 was increased upon RSV infection, suggesting IL-17 might augment renal damage by contributing to the persistency of RSV after RSV infection.

In this study, we found that there were no significant differences in the levels of blood urea nitrogen and serum creatinine between the groups, suggesting that the glomerular filtration rate was normal after RSV infection. Proteinuria was increased after RSV infection, which might be due to damage of the glomerular basement membrane and protein leaking through the glomeruli. However, urea nitrogen and creatinine are also discharged through the urine. Proteinuria is a clinical symptom of kidney disease, while blood urea nitrogen/creatinine are markers of renal function and are often elevated during the end-stage of chronic renal disease or during the period of the mostly glomerular dysfunction. Proteinuria is more sensitive than BUN/creatinine for evaluating renal disease. Furthermore, for children with simple nephrotic syndrome, renal function is typically normal, which is in accordance with our results. These results also conform with our previous study (Liu et al., 2006).

The present study had some potential limitations. First, we examined lymphocytes in the peripheral blood, but not in the kidney or in the draining lymphoid tissues, thus could not directly verify the role of CD8^+^ lymphocytes on the renal damage induced by RSV. Secondly, the DCs we studied were cultured and differentiated from bone marrow and examined for the expression of OX62 to evaluate the number of DCs. The addition of another marker, such as CD11, would have strengthened this analysis. On the other hand, although they could be influenced by RSV infection, if the DCs cultured and differentiated from PBMCs or renal DCs were studied, these results might be more convincing. Third, despite that levels of cholesterol and triglyceride are not essential criteria for diagnosing simple nephrotic syndrome, cholesterol, and triglyceride levels should have been examined in this study.

In summary, our study showed that RSV infection could induce renal injury, and reinfection could aggravate renal injury, which might be due to both direct renal damage and immune dysregulation. These results elucidate the mechanism of infection-induced onset and relapse of NS.

## Author contributions

ZW is responsible for project design and experiments implementation; SZ is responsible for clinical experiments and writing the article; LH is responsible for immunology experiments and writing the article; LZ and YG are responsible for the establishment of animal model; LD and RJ is responsible for data analysis.

### Conflict of interest statement

The authors declare that the research was conducted in the absence of any commercial or financial relationships that could be construed as a potential conflict of interest.
